# Transcription Factor Hb9 Is Expressed in Glial Cell Lineages in the Developing Mouse Spinal Cord

**DOI:** 10.1523/ENEURO.0214-22.2022

**Published:** 2022-11-02

**Authors:** Sunjay Letchuman, Ashley Tucker, Diego Miranda, Robert L. Adkins, Miriam Aceves, Valerie Dietz, Vipin Jagrit, Amy Leonards, Young il Lee, Jennifer N. Dulin

**Affiliations:** 1 Department of Biology, Texas A&M University, College Station, TX 77843; 2 Mays Business School, Texas A&M University, College Station, TX 77843; 3 Texas A&M Institute for Neuroscience, Texas A&M University, College Station, TX 77843; 4 Department of Pharmacology and Therapeutics, University of Florida College of Medicine, Gainesville, FL 32610; 5 Myology Institute, University of Florida College of Medicine, Gainesville, FL 32610

**Keywords:** astrocytes, Cre recombinase, Hb9, oligodendrocytes, spinal motor neurons

## Abstract

Hb9 (*Mnx1*) is a transcription factor described as a spinal cord motor neuron (MN)-specific marker and critical factor for the postmitotic specification of these cells. To date, expression of Hb9 in other cell types has not been reported. We performed a fate-mapping approach to examine distributions of Hb9-expressing cells and their progeny (“Hb9-lineage cells”) within the embryonic and adult spinal cord of Hb9^cre^;Ai14 mice. We found that Hb9-lineage cells are distributed in a gradient of increasing abundance throughout the rostrocaudal spinal cord axis during embryonic and postnatal stages. Furthermore, although the majority of Hb9-lineage cells at cervical spinal cord levels are MNs, at more caudal levels, Hb9-lineage cells include small-diameter dorsal horn neurons, astrocytes, and oligodendrocytes. In the peripheral nervous system, we observed a similar phenomenon with more abundant Hb9-lineage Schwann cells in muscles of the lower body versus upper body muscles. We cultured spinal cord progenitors *in vitro* and found that gliogenesis was increased by treatment with the caudalizing factor FGF-8B, while glial tdTomato expression was increased by treatment with both FGF-8B and GDF-11. Together, these observations suggest that early and transient expression of Hb9 in spinal cord neural progenitors may be induced by caudalizing factors such as FGF and GDF signaling. Furthermore, our work raises the possibility that early Hb9 expression may influence the development of spinal cord macroglia and Schwann cells, especially at caudal regions. Together, these findings highlight the importance of using caution when designing experiments using Hb9^cre^ mice to perform spinal cord MN-specific manipulations.

## Significance Statement

The transcription factor Hb9 is key in postmitotic specification of spinal cord motor neurons (MNs), and thought to be expressed specifically in this cell population. We performed fate-mapping experiments and found that Hb9-lineage cells are not restricted to motor neurons, but also include spinal cord macroglia and Schwann cells. Strikingly, Hb9-lineage cells are present in an increasing rostrocaudal gradient in the CNS. Proportions of Hb9-lineage astrocytes in cultures of spinal cord progenitors could be manipulated by treatment with caudalizing factors FGF-8B and GDF-11. These findings highlight an interesting developmental phenomenon in which there is an increasing rostrocaudal gradient of molecularly-defined cell types. Further, these findings urge caution for researchers using Hb9^cre^ mice to study spinal motor neurons in isolation.

## Introduction

Hb9 (*Mnx1*) is a well-established marker for motor neurons (MNs) within the developing CNS (Tanabe et al., 1998; SK Lee et al., 2004; Nakano et al., 2005). Its role in the consolidation of MN identity includes maintaining normal MN migratory patterns and proper muscle innervation (Pfaff et al., 1996; Arber et al., 1999). In mice, Hb9 expression has first been detected at embryonic day (E)9.5, which coincides with the emergence of the first postmitotic MNs in the spinal cord (Arber et al., 1999; Caldeira et al., 2017). In Hb9-mutant mice, migratory patterns of MNs are disrupted, MN differentiation is disturbed, motor axons project abnormally, and the phrenic nerve fails to form–resulting in stillbirth (Thaler et al., 1999; Yang et al., 2001). However, differentiation of MN progenitors within the spinal cord into somatic and visceral MNs remains constant (Arber et al., 1999; Stifani, 2014). Mutations in the human *HB9* gene cause disruptions in dorsal-ventral patterning resulting in a congenital malformation, characterized by significant sacral defects, called Currarino syndrome (Dworschak et al., 2021).

Expression of Hb9 in the spinal cord of the embryonic chick has been shown to induce MN differentiation while suppressing the differentiation of V2 interneurons, which emerge from an adjacent progenitor domain (Thaler et al., 1999). Researchers studying avian embryonic development have found that Hb9 expression in MNs is largely regulated by the expression of the morphogen sonic hedgehog (Shh; Kahane and Kalcheim, 2020). Hence, Hb9 is widely considered to be a major regulatory switch that induces spinal cord MN cell fate. However, to date there have not been any published reports of Hb9 expression in other cell types within the spinal cord, except for a population of excitatory Shox2 non-V2a interneurons involved in rhythm generation (Caldeira et al., 2017).

To investigate Hb9 expression in early embryonic development, we performed fate mapping experiments using Hb9^cre^;Ai14 mice. Heterozygous Hb9^cre^ mice, which express Cre recombinase in place of one copy of the Hb9 gene (Arber et al., 1999; Yang et al., 2001), were crossed with Ai14 (Rosa-CAG-LSL-tdTomato-WPRE) mice, which carry a loxP-flanked STOP cassette upstream of the tdTomato reporter gene (Madisen et al., 2010). In the F1 generation of the Hb9^cre^;Ai14 cross, 50% of progeny inheriting the Cre allele will have the STOP cassette deleted in the Cre-expressing tissue(s), resulting in robust tdTomato fluorescence. Hence, the Hb9^cre^;Ai14 mouse serves as a robust reporter for Hb9 fate mapping because all of the cells with endogenous Hb9 expression, and their progeny, are permanently labeled with tdTomato.

Using this approach, we found that Hb9-lineage cells are distributed in an increasing gradient along the rostrocaudal axis in the embryonic and adult spinal cord. Hb9-lineage cells were not restricted to MNs, but rather included astrocytes and oligodendrocytes in the adult spinal cord, especially at caudal levels. In the peripheral nervous system, terminal Schwann cells were labeled with the Hb9-lineage reporter, especially at neuromuscular junctions (NMJs) in muscles of the hindlimbs. This rostrocaudal gradient was apparent as early as E9.5, where some neural progenitors in the neural tube exhibited nuclear Hb9 localization. *In vitro*, cultured spinal cord neural progenitors expressed tdTomato in neurons and glia, and proportions of Hb9-lineage astrocytes could be manipulated by treatment with caudalizing morphogens.

## Materials and Methods

### Ethics statement

Animal studies were performed in stringent compliance with the NIH *Guidelines for Animal Care and Use of Laboratory Animals*, and the Society for Neuroscience Policies on the Use of Animals and Humans in Neuroscience Research. All animal procedures were performed in accordance with the Texas A&M University Institutional Animal Care and Use Committee. All efforts were made to minimize pain and distress.

### Animals

Both male and female mice were used for this study, including C57BL/6 mice (#000664, The Jackson Laboratory), Ai14 (B6.Cg-*Gt(ROSA)26Sor^tm14(CAG-tdTomato)Hze^*/J, #007914, The Jackson Laboratory), and Hb9^cre^ (B6.129S1-*Mnx1^tm4(cre)Tmj^*/J, #006600, The Jackson Laboratory). Animals ranging from age postnatal day (P)0 to 12 weeks old were used for experiments. Animals had free access to food and water throughout the study and were group-housed (up to five littermates per cage) in standard Plexiglas cages on a 12/12 h light/dark cycle (light cycle = 6 A.M. to 6 P.M.).

### Embryo generation and neural progenitor cell (NPC) isolation

For generation of Hb9^cre^;Ai14 progeny, Hb9^cre^ males were used as sires and Ai14 females were used as dams. Mouse embryos were generated through timed mating between heterozygous Hb9^cre^ males and Ai14(RCL-tdT)-D females. Adult female mice received intraperitoneal injections of luteinizing hormone-releasing hormone (5 I.U.; Sigma-Aldrich #L-4513) and 4 d later, females were paired with males overnight. When possible, pregnancy was confirmed by palpation of the abdomen 12 d later. Upon embryo collection, Cre^+^ embryos were visualized using a fluorescent flashlight with a green excitation filter (NIGHTSEA). Embryonic spinal cords were dissected in ice-cold HBSS. For *in vitro* studies, spinal cords were cut transversely at ∼4.0–4.5 mm from the rostral end to separate the anterior and posterior segments. In this way, the “anterior” segment included the cervical and upper half of the thoracic cord, and the “posterior” segment included the lumbar and lower half of the thoracic cord.

For neural progenitor cell (NPC) isolation, anterior and posterior spinal cord segments were digested separately in 0.125% trypsin at 37°C for 8–10 min (≤10 spinal cords were pooled for a single cell preparation). Fetal bovine serum (10% in DMEM) was then added at a 10:1 volume ratio to halt the trypsinization reaction, and spinal cords were centrifuged at 600 RCF for 2 min. Supernatant was removed and tissue was gently triturated in Neurobasal Medium + 2% B27 (NBM/B27) until cell suspension appeared milky and homogeneous (∼20 passes through a P1000 pipette tip). Cell suspensions were then centrifuged at 600 RCF for 2 min. Supernatant was removed and cells were resuspended in 2–3 ml of NBM/B27, then passed through a 40-μm cell strainer. Cell viability was assessed by trypan blue exclusion and confirmed to be >95% in all cases. Cells were then plated in 48-well culture plates at 1 million cells per well, and cultured for 10 d with once daily media changes. Media was removed, and cells were fixed with 2% PFA for 20 min at room temperature, then washed and stored in PBS until immunocytochemistry was performed.

### Drug treatment

For drug treatment experiments, only anterior spinal cord neural progenitor cells were used. Either CHIR 99 021 (2 μm; Tocris #4423, batch #13), recombinant human/murine FGF-8b (200 ng/ml; Peprotech #100–25, lot #0220161), recombinant human/murine/rat GDF-11 (50 ng/ml; Peprotech #120-11, lot #0314295), or vehicle (0.1% DMSO) was added into 250 μl of media beginning immediately after plating and continuing once daily for 7 d. Media were removed, and cells were fixed with 2% paraformaldehyde (PFA) for 20 min at room temperature, then washed and stored in PBS until immunocytochemistry was performed.

### Spinal cord immunohistochemistry

Animals were euthanized by anesthesia overdose and transcardially perfused with 30 ml of 0.1 m phosphate buffer + 0.9% NaCl (PB) followed by 30 ml of 4% PFA in 0.1 m PB. Spinal columns were removed and postfixed in 4% PFA in 0.1 m PB overnight at 4°C, then cryopreserved in 30% sucrose in 0.1 m PB at 4°C for at least 3 d before cryosectioning. For fixation of embryos, the pregnant mother was perfused as above, then embryos were removed and postfixed in 4% PFA for 30 min to 2 h at room temperature. Embryos were then cryopreserved in sucrose for at least 3 d before cryosectioning. Adult and embryonic spinal cord tissue was embedded in Tissue-Tek OCT compound (VWR) and frozen on dry ice. Spinal cord tissue was cryosectioned in the transverse or sagittal plane to a thickness of 30 μm. Sections were either collected into a 24-well plate filled with storage solution or directly mounted on slides and stored at −20°C.

Either a 1-in-6 or a 1-in-12 tissue series was used for immunohistochemistry experiments. Sections were washed in tris-buffered saline (TBS) three times for 10 min each, then blocked in TBS containing 5% donkey serum (Lampire Biological Laboratories, #7332100) and 0.25% Triton X-100 (Sigma-Aldrich) for 1 h at room temperature. Sections were then incubated with primary antibodies (Table 1) diluted in blocking solution overnight at 4°C. The next day, sections were washed in TBS three times for 10 min each, then incubated with Alexa Fluor-conjugated secondary antibodies (Jackson ImmunoResearch) diluted 1:1000 in blocking solution for 2 h at room temperature. Finally, sections were washed in TBS three times for 10 min each, with the final wash containing DAPI (5 μg/ml, Sigma-Aldrich, D9542). Free-floating sections were mounted to gelatin-coated slides, air-dried, rinsed in distilled water, and coverslipped with Mowiol mounting medium.

**Table 1:** Primary antibodies used in this study

Antibody	Catalog #	RRID	Dilution
Goat anti-ChAT	Genetex #GTX82725	AB_11162364	1:400
Chicken anti-GFAP	Encor Bio #CPCA-GFAP	AB_2109953	1:1500
Mouse anti-Hb9	DSHB #81.5C10	AB_2145209	1:50
Goat anti-mCherry	Sicgen #AB0040	AB_2333092	1:2000
Guinea pig anti-NeuN	Millipore #ABN90	AB_11205592	1:3000
Mouse anti-neurofilament (NF-M)	DSHB #2H3	AB_531793	1:500
Rabbit anti-Olig2	Millipore #AB9610	AB_570666	1:200
Mouse anti-Pax7	DSHB #AB528428	AB_528428	1:50
Rabbit anti-RFP	Abcam #62341	AB_945213	1:500
Rabbit anti-S100B	Dako (now Agilent: Z031129-2)	AB_2315306	1:500
Mouse anti-synaptic vesicle glycoprotein 2A	DSHB #SV2	AB_2315387	1:500
Rabbit anti-Sox9	Abcam #185966	AB_2728660	1:2000

### Fluorescent imaging of neuromuscular junctions

Muscles for whole-mount labeling of neuromuscular junctions (NMJs) were prepared and immunostained as described previously (YI Lee et al., 2016; YI Lee, 2019). Briefly, the animals were transcardially perfused with PBS, pH 7.4. The extensor digitorum longus, extensor hallucis longus, diaphragm, soleus, sternomastoid and triangularis sterni muscles were dissected and fixed in 4% phosphate-buffered PFA, pH 7.4 for 20 min at room temperature and rinsed in three changes, 5 min each, of PBS. To label surface nicotinic acetylcholine receptor (AChR) at NMJs, fixed muscles were incubated with fluorescently-labeled α-bungarotoxin (α-BTX; a snake toxin which binds specifically and with high affinity to AChR; 1:500; Invitrogen) before permeabilization. The presynaptic nerve terminals were labeled with a mixture of monoclonal antibodies (mAbs) to neurofilament and synaptic vesicles (2H3 and SV2, respectively). Schwann cells were labeled with a rabbit polyclonal antibody against S100B.

### Immunocytochemistry

Fixed cells were washed with PBS and blocked with 5% donkey serum in TBS for 1 h at room temperature, followed by incubation with primary antibodies (Table 1) diluted in blocking solution for 1.5 h at room temperature. Cells were then washed in TBS three times for 5 min each, incubated in Alexa Fluor-conjugated secondary antibodies (Jackson ImmunoResearch) diluted 1:1000 in blocking solution for 1 h at room temperature, and then incubated in 5 μg/ml DAPI for 10 min. Cells were washed in TBS three times for 5 min each, and stored in TBS at 4°C before imaging.

### Image acquisition

Samples labeled with fluorescent dyes were imaged in a dark room. Images were acquired using the same acquisition settings across all samples for each immunohistochemical label. Slides were imaged using a Nikon Eclipse upright fluorescent microscope equipped with a Prior Scientific XY motorized stage and a Zyla 4.2 PLUS monochrome camera (Andor), and cells were imaged using a Nikon Eclipse Ti2 inverted fluorescent microscope. Nikon NIS-Elements software was used for image acquisition and XY stitching. Images were captured with a 10× or 20× magnification objective. Confocal microscope was performed using an Olympus FV1000. To generate representative images (not used for quantification), the Extended Depth of Focus module in NIS-Elements was sometimes used to create focused images from Z-stacks. Images were exported as 8-bit TIFF files for analysis. For NMJ imaging, images were acquired using a Zeiss LSM 780 confocal system or a Leica DMR epifluorescence microscope equipped with a Hamamatsu cooled CCD camera controlled by a Macintosh computer with iVision software (BioVision Technologies).

### Image analysis

All image analysis was performed using ImageJ software. Images of mCherry, NeuN, Olig2, and Sox9 immunoreactivity were thresholded using the ImageJ Auto Local Threshold function with Phansalkar’s thresholding method. Watershed was applied to binary images and the Analyze Particles function was used to count the total number of cells in the entire image [no regions of interest (ROIs) were drawn]. Automated cell counting methods were always validated by manual counts for two images within each batch; two independent experimenters hand-counted cells in a blinded fashion, then hand counts were compared with results generated with the automated cell counting macro. We found that interexperimenter counts were 99% accurate and >95% consistent with automated counts.

#### Quantification of tdTomato^+^ cells in dorsal and ventral spinal cord

**Figure 1.**
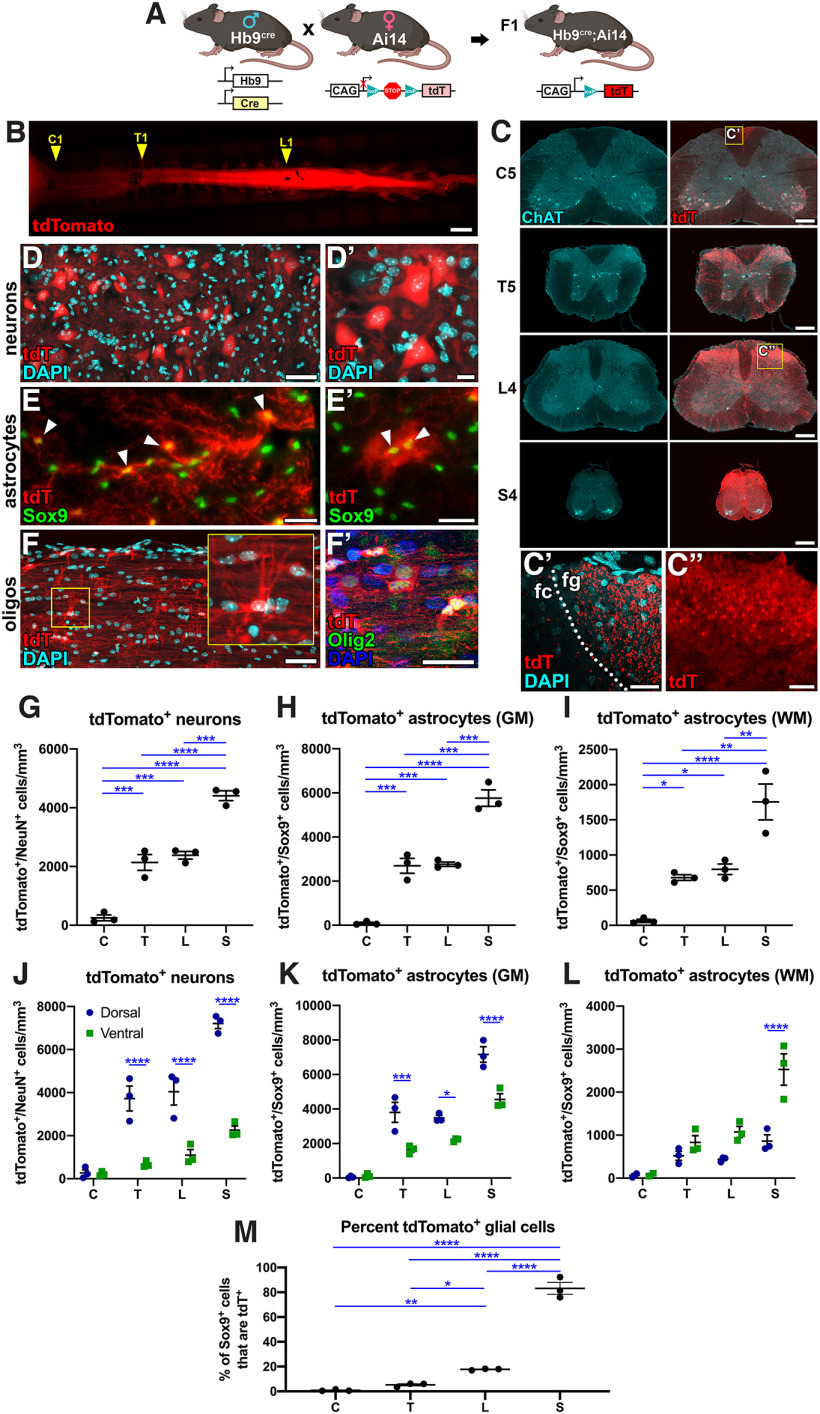
tdTomato is expressed in glial cells of the adult Hb9^cre^;Ai14 mouse in an increasing rostrocaudal gradient. ***A***, Cartoon depicting the breeding scheme used to generate mice in this study. Hb9^cre^ male sires were mated to Ai14 female dams to produce Hb9^cre^;Ai14 F1 offspring. ***B***, Gross image of whole spinal cord from a P28 Hb9^cre^;Ai14 mouse. Black markings and arrowheads indicate the position of C1, T1, and L1 spinal levels. ***C***, Transverse sections of cervical (C5), thoracic (T5), lumbar (L4), and sacral (S4) spinal cord labeled for choline acetyltransferase (ChAT) and tdTomato (tdT). Images are from a P28 Hb9^cre^;Ai14 mouse. ***C’***, Inset shows tdTomato^+^ axons in the fasciculus gracilis (fg) but not fasciculus cuneatus (fc) of the cervical spinal cord. ***C’’***, Inset shows small-diameter tdTomato^+^ neurons in the lumbar dorsal horn. ***D–F***, High-magnification images of sagittal sections from the thoracic spinal cord of a P28 Hb9^cre^;Ai14 mouse. ***D***, ***D’***, tdTomato^+^ cells with neuronal morphology. ***E*, *E’***, tdTomato^+^ cells with astroglial cell morphology that express glial cell marker Sox9 (arrowheads). ***F***, tdTomato^+^ cells with oligodendrocyte-like morphology. ***F’***, Some tdTomato^+^ cells exhibit nuclear expression of Olig2. ***G–I***, Quantification of the number of tdTomato^+^ (***G***) neurons, (***H***) gray matter (GM) astrocytes, and (***I***) white matter (WM) astrocytes at cervical (C5), thoracic (T5), lumbar (L4), and sacral (S4) spinal segments. ***J–L***, Quantification of the numbers of tdTomato^+^ (***J***) neurons, (***K***) gray matter astrocytes, and (***L***) white matter astrocytes in the dorsal or ventral halves of each spinal cord tissue section. ***M***, Quantification of the percent of total Sox9^+^ glial cells that express tdTomato at each spinal level. Detailed descriptions of statistical analyses are provided in Table 1. *p < 0.05, **p < 0.01, ***p < 0.001, ****p < 0.0001. All data are mean ± SEM *N* = 3 per group. Scale bars = 2 mm (***B***), 250 μm (***C***), 100 μm (***D***, ***F***), 50 μm (***C’’***), and 25 μm (***C’***, ***D’***, ***E***, ***E’***, ***F’***).

ROIs were drawn for individual tdTomato^+^/NeuN^+^ cells (Fig. 1*G*,*J*) or tdTomato^+^/Sox9^+^ cells (Fig. 1*H*,*I*,*K*,*L*) in spinal cord tissue sections. A horizontal line was drawn onto each image through the central canal, such that the line separated the dorsal and ventral halves of the spinal cord. Individual cell ROIs were thereby determined to be either “dorsal” or “ventral.”

#### Quantification of Schwann cells

Within the confines of NMJs, the Hb9^Cre^-driven expression of tdTomato was deemed to be present in Schwann cells when its expression mirrors S100B fluorescence in addition to its accumulation in Schwann cell somata, but restricted to motor axon terminals when no Schwann cell labeling is observed.

#### Quantification of cell cluster size

Regions of interest were manually drawn around contiguous clusters of DAPI^+^ cells in which all cells were touching each other, and the total area of each cluster was measured in ImageJ. ROIs were drawn around every cluster in all images to avoid quantification bias.

### Statistical analysis

GraphPad Prism 8 (GraphPad Software) was used to perform statistical analysis. The experimental design was random. All data are presented as mean ± SEM. Statistical significance was defined as *p* < 0.05. Detailed statistical methods are included in Table 2.

**Table 2:** Detailed description of statistical analyses used in this study

Figure	Parameter	Sample size	Statistical test	Significance level
1*G*	tdTomato^+^ neurons in P28 spinal cord	*N* = 3 mice per group	Ordinary one-way ANOVA with Tukey’s multiple comparisons test	*F*_(3,8)_ = 90.22, *p* < 0.0001 C vs T: *p* = 0.0003 C vs L: *p* = 0.0001 C vs S: *p* < 0.0001 T vs S: *p* < 0.0001 L vs S: *p* = 0.0002
1*H*	tdTomato^+^ gray matter astrocytes in P28 spinal cord	*N* = 3 mice per group	Ordinary one-way ANOVA with Tukey’s multiple comparisons test	*F*_(3,8)_ = 81.54, *p* < 0.0001 C vs T: *p* = 0.0004 C vs L: *p* = 0.0004 C vs S: *p* < 0.0001 T vs S: *p* = 0.0001 L vs S: *p* = 0.0002
1*I*	tdTomato^+^ white matter astrocytes in P28 spinal cord	*N* = 3 mice per group	Ordinary one-way ANOVA with Tukey’s multiple comparisons test	*F*_(3,8)_ = 26.84, *p* = 0.0002 C vs T: *p* = 0.0486 C vs L: *p* = 0.0206 C vs S: *p* < 0.0001 T vs S: *p* = 0.0022 L vs S: *p* = 0.0045
1*J*	tdTomato^+^ neurons in P28 spinal cord (dorsal vs ventral)	*N* = 3 mice per group	Two-way ANOVA with Sidak’s multiple comparisons test	*F*_(1,16)_ = 133.7, *p* < 0.0001 (source of variation: dorsal vs ventral) T dorsal vs T ventral: *p* < 0.0001 L dorsal vs L ventral: *p* < 0.0001 S dorsal vs S ventral: *p* < 0.0001
1*K*	tdTomato^+^ gray matter astrocytes in P28 spinal cord (dorsal vs ventral)	*N* = 3 mice per group	Two-way ANOVA with Sidak’s multiple comparisons test	*F*_(1,16)_ = 51.32, *p* < 0.0001 (source of variation: dorsal vs ventral) T dorsal vs T ventral: *p* = 0.0004 L dorsal vs L ventral: *p* = 0.0294 S dorsal vs S ventral: *p* < 0.0001
1*L*	tdTomato^+^ white matter astrocytes in P28 spinal cord (dorsal vs ventral)	*N* = 2–3 mice per group)	Two-way ANOVA with Sidak’s multiple comparisons test	*F*_(1,14)_ = 25.83, *p* = 0.0002 (source of variation: dorsal vs ventral) S dorsal vs S ventral: *p* < 0.0001
1*M*	Percent of all Sox9^+^ cells that express tdTomato in P28 spinal cord	*N* = 3 mice per group	Ordinary one-way ANOVA with Tukey’s multiple comparisons test	*F*_(3,8)_ = 242.9, *p* < 0.0001 C vs L: *p* = 0.0055 C vs S: *p* < 0.0001 T vs L: *p* = 0.0289 T vs S: *p* < 0.0001 L vs S: *p* < 0.0001
2*C*	tdTomato^+^ Schwann cells at NMJs in P28 mice	*N* = 3–4 per group (between 110 and 344 NMJs quantified per individual muscle)	Ordinary one-way ANOVA with Bonferroni’s multiple comparisons test	*F*_(5,14)_ = 3.392, *p* = 0.0324 TS vs SOL: *p* = 0.0306 TS vs EDL: *p* = 0.0539
7*D*	tdTomato^+^ neurons *in vitro*	*N* = 12 wells per group	Unpaired *t* test	*t* = 25.15, df = 22 *p* < 0.0001
7*E*	tdTomato^+^ Sox9^+^ cells *in vitro*	*N* = 6 wells per group	Unpaired *t* test	*t* = 8.772, df = 10 *p* < 0.0001
7*F*	tdTomato^+^ Olig2^+^ cells *in vitro*	*N* = 6 wells per group	Unpaired *t* test	*t* = 8.287, df = 10 *p* < 0.0001
8*C*	Cluster size *in vitro* + drug treatment	*N* = 6 wells per group (between 153 and 319 clusters quantified per well)	Ordinary one-way ANOVA with Dunnett’s multiple comparisons test	*F*_(4,1096)_ = 363.7, *p* < 0.0001 VEH vs CHIR: *p* < 0.0001 VEH vs FGF: *p* < 0.0001 VEH vs FGF+GDF: *p* < 0.0001
8*E*	Percent neurons *in vitro* + drug treatment	*N* = 6 wells per group	Ordinary one-way ANOVA with Dunnett’s multiple comparisons test	*F*_(4,25)_ = 8.967, *p* = 0.0001 VEH vs FGF: *p* = 0.0003
8*F*	Percent astrocytes *in vitro* + drug treatment	*N* = 6 wells per group	Ordinary one-way ANOVA with Dunnett’s multiple comparisons test	*F*_(4,25)_ = 36.21, *p* < 0.0001 VEH vs FGF: *p* < 0.0001 VEH vs FGF+GDF: *p* = 0.0027
8*G*	Percent tdTomato^+^ cells *in vitro* + drug treatment	*N* = 6 wells per group	Ordinary one-way ANOVA with Dunnett’s multiple comparisons test	*F*_(4,25)_ = 2.502, *p* = 0.0680 No significant differences
8*H*	Percent tdTomato^+^ neurons *in vitro* + drug treatment	*N* = 6 wells per group	Ordinary one-way ANOVA with Dunnett’s multiple comparisons test	*F*_(4,25)_ = 3.596, *p* = 0.0190 No significant differences
8*I*	Percent tdTomato^+^ astrocytes *in vitro* + drug treatment	*N* = 6 wells per group	Ordinary one-way ANOVA with Dunnett’s multiple comparisons test	*F*_(4,25)_ = 6.630, *p* = 0.0009 VEH vs CHIR: *p* = 0.0244 VEH vs FGF+GDF: *p* = 0.0002

## Results

### tdTomato labels neurons and glial cells in increasing rostrocaudal abundance in the adult and embryonic Hb9^cre^;Ai14 mouse spinal cord

**Figure 2.**
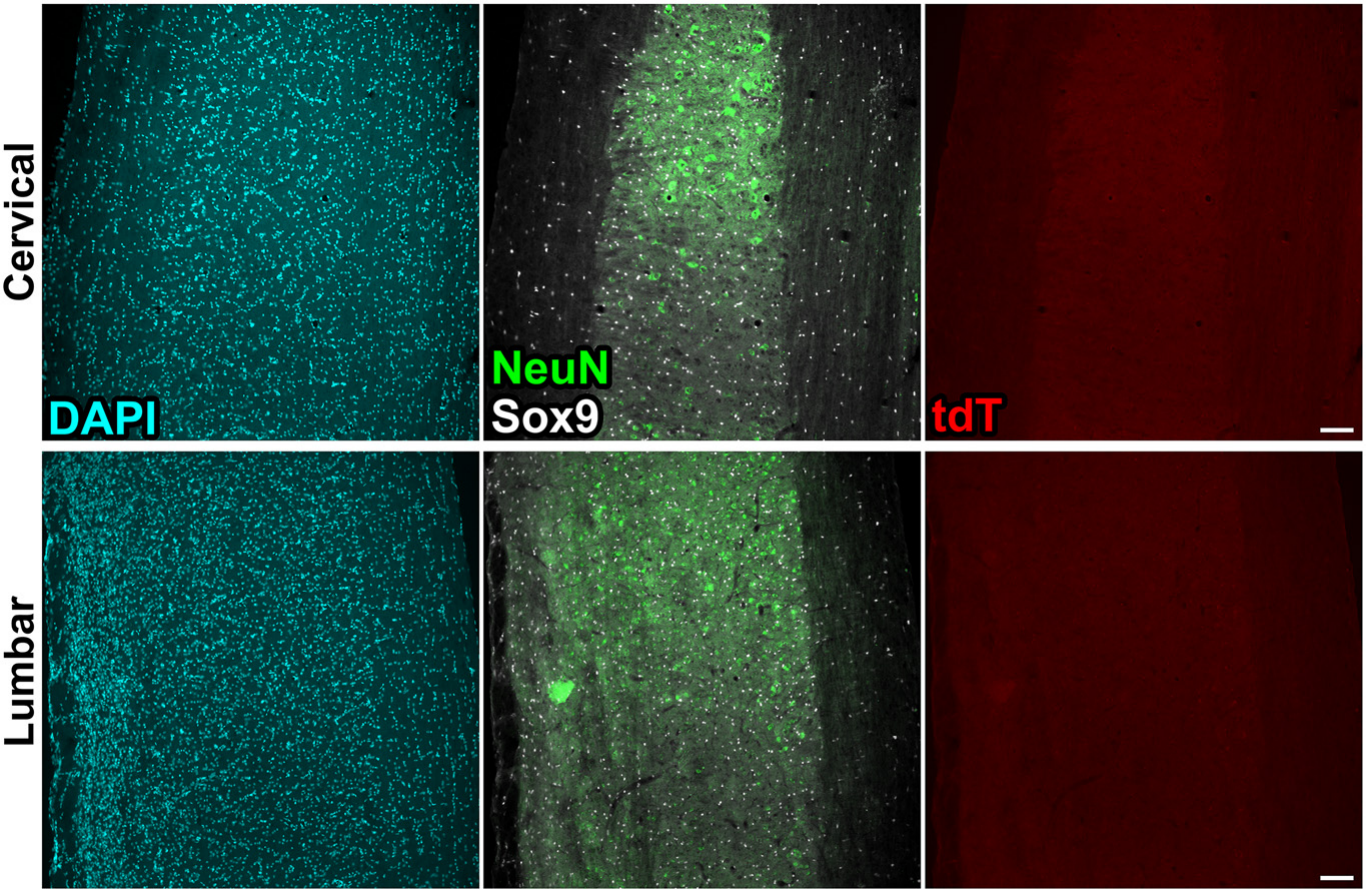
tdTomato (tdT) is not expressed in Ai14 spinal cords in the absence of Cre recombinase. Images of sagittal spinal cord sections from cervical (C4–C6; top row) and lumbar (L2–L6; bottom row) spinal cords of adult Ai14 mice. Neurons are labeled with NeuN and astrocyte nuclei are labeled with Sox9. Scale bars = 100 μm.

We sought to generate transgenic mice with fluorescent reporter expression in spinal cord motor neurons. Because of the well-described role of Hb9 in consolidating MN identity and its use as a MN-specific marker (Arber et al., 1999; Nakano et al., 2005; Amoroso et al., 2013), we crossed male Hb9^cre^ mice with female Ai14 mice to generate F1 progeny with the Hb9^cre^;Ai14 genotype (Fig. 1*A*). We expected these mice to express the tdTomato (tdT) reporter in all Hb9^+^ MNs as well as Hb9^+^ interneurons (Wilson et al., 2005; Caldeira et al., 2017). Surprisingly, on inspection of spinal cord tissue of Hb9^cre^;Ai14 mice, we found that tdT expression was visible in an increasing rostrocaudal gradient, with low levels of fluorescence in the rostral spinal cord and high fluorescence levels in the caudal cord (Fig. 1*B*). We analyzed reporter expression in Ai14 mice (without Cre), and found a complete absence of tdTomato expression (Fig. 2), indicating that reporter expression is Cre-dependent, as expected. Transverse sections of spinal cord tissue were inspected at cervical, thoracic, lumbar, and sacral levels to examine patterns of tdT expression. We found that in the cervical spinal cord, tdT expression was largely restricted to choline acetyltransferase (ChAT)-expressing MNs, with the exception of very few tdT^+^ cells with glial morphology as well as tdT^+^ axons in the fasciculus gracilis (Fig. 1*C****’***). Interestingly, we did not observe tdT^+^ axons in the fasciculus cuneatus, which is composed of ascending axons derived from sensory neurons at cervical levels (Fig. 1*C****’***). In contrast, at more caudal levels of the spinal cord, tdT expression was not mostly restricted to MNs; abundant tdT^+^ cells with glial morphology were distributed throughout the white and gray matter of the spinal cord (Fig. 1*C*). On closer inspection of tdTomato^+^ cells, we found that they expressed markers of neurons, astrocytes, and oligodendrocytes (Fig. 1*D–F*). We therefore quantified the numbers of tdT^+^ neurons and glial cells at cervical, thoracic, lumbar, and sacral spinal levels (Fig. 1*G–I*). Whereas only MNs were labeled in the cervical spinal cord gray matter (252 ± 101 tdT^+^/NeuN^+^ cells/mm^3^), there were significantly more tdT^+^/NeuN^+^ neurons in the dorsal horn of the thoracic, lumbar, and sacral spinal cord (T: 2140 ± 269; L: 2380 ± 130; S: 441 ± 168 cells/mm^3^; Fig. 1*C’’*,*G*). In the spinal cord gray matter, significantly fewer tdT^+^ astrocytes were present in the cervical spinal cord (92.4 ± 46.8 tdT^+^/Sox9^+^ cells/mm^3^) compared with all other levels (T: 2690 ± 337; L: 2770 ± 98.6; S: 5760 ± 371 cells/mm^3^), with sacral levels containing more tdT^+^ astrocytes than more rostral levels (Fig. 1*H*). In the spinal cord white matter, there were significantly more tdT^+^ astrocytes in the sacral cord (1760 ± 255 tdT^+^/Sox9^+^ cells/mm^3^) than all other levels (C: 71.4 ± 36.6; T: 679 ± 40.3; L: 797 ± 74.9 cell/mm^3^; Fig. 1*I*).

We also compared the numbers of tdT^+^ cells that were either located in the dorsal or the ventral halves of the spinal cord. We found that there were significantly greater numbers of tdT^+^ neurons in the dorsal gray matter compared with the ventral gray matter at thoracic, lumbar, and sacral levels (Fig. 1*J*). Similarly, there were significantly greater numbers of tdT^+^ astrocytes in the dorsal gray matter versus the ventral gray matter at these caudal spinal levels (Fig. 1*K*). In contrast, white matter astrocytes showed the opposite effect; there were significantly greater numbers of tdT^+^ astrocytes in the ventral white matter at sacral levels (Fig. 1*L*). We also quantified the percentages of Sox9^+^ glial cells that expressed tdTomato at each spinal level, and found that the sacral spinal cord had the highest percentage of tdT^+^ glia (83.2 ± 4.80%), significantly greater than all other levels (C: 0.818 ± 0.403%; T: 5.22 ± 0.884%; L: 17.7 ± 0.399%; Fig. 1*M*). Together, these data reveal that neurons and glial cells derived from Hb9^+^ lineages, are distributed in an increasing gradient along the spinal cord rostrocaudal axis. We found that none of these cells had detectable Hb9 immunoreactivity in the adult spinal cord (data not shown), so we refer to these as “Hb9-lineage cells.”

**Figure 3.**
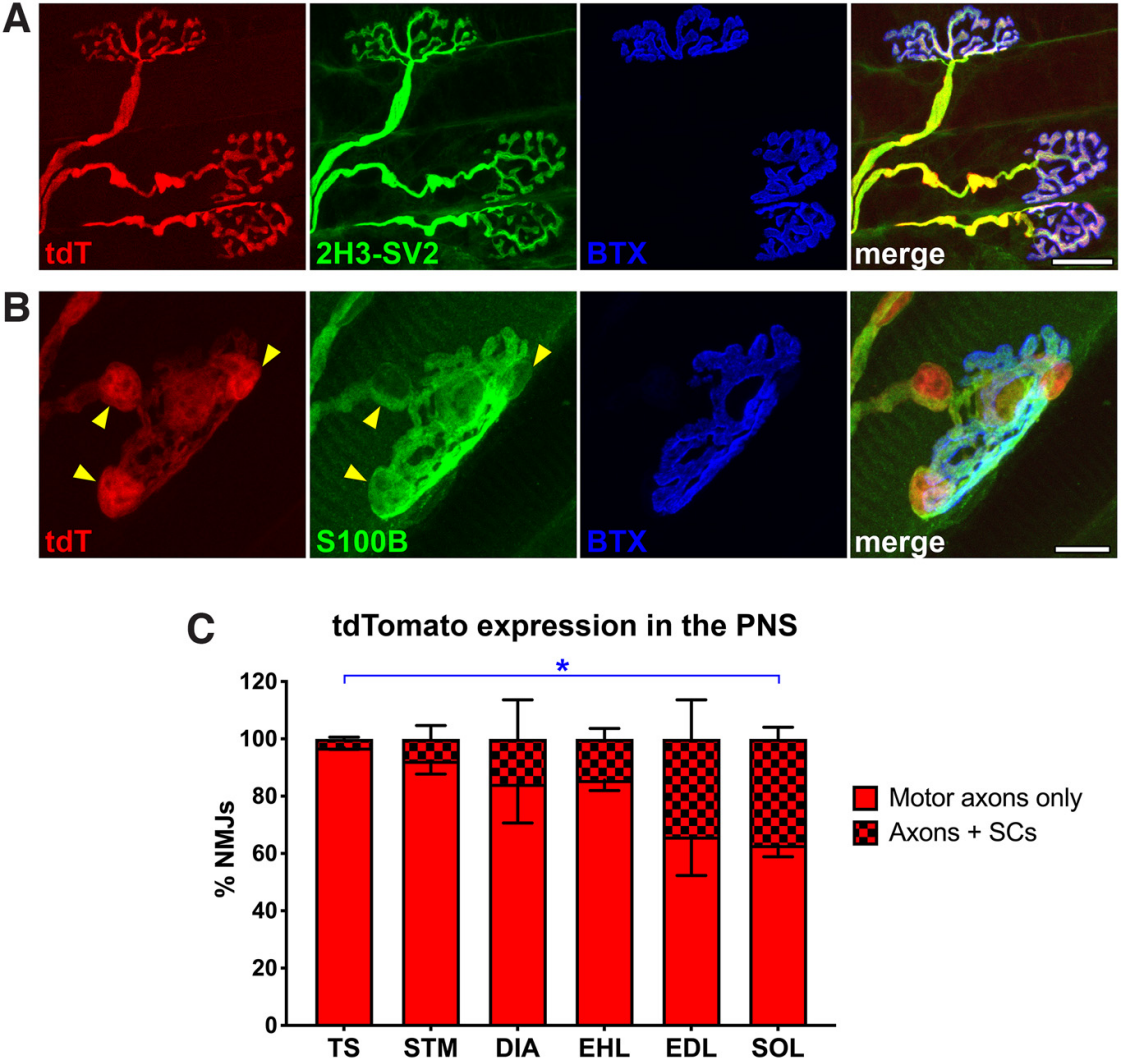
Schwann cells express tdTomato in Hb9^cre^;Ai14 mice. ***A***, Representative image of tdTomato^+^ motor axons immunolabeled against neurofilament and synaptic vesicle glycoprotein 2A (2H3-SV2); acetylcholine receptors at the neuromuscular junction are labeled with α-bungarotoxin (α-BTX). ***B***, Some Schwann cells (S100B) at the neuromuscular junction also express tdTomato (arrowheads). ***C***, Quantification of the number of neuromuscular junctions that exhibited tdTomato expression in motor axons only (solid red), or both motor axons and Schwann cells (red checkered bar), in the triangularis sterni (TS), sternomastoid (STM), diaphragm (DIA), extensor hallucis longus (EHL), extensor digitorum longus (EDL), and soleus (SOL). *p < 0.05. All data are mean ± SEM *N* = 3–4 per group. Scale bars = 20 μm (***A***) and 10 μm (***B***).

The Hb9^cre^ mouse has previously been used to target motor neurons, either for their deletion or gene inactivation, in examining innervation of postsynaptic skeletal muscle fibers (Yang et al., 2001; Chipman et al., 2014). In light of the above observation that glial populations within spinal cord are Hb9-lineage cells, we examined the possibility of similar glial expression in the peripheral nervous system. We examined patterns of tdTomato expression at NMJs within six different muscles along the rostrocaudal axis from postnatal Hb9^cre^;Ai14 mice. Although tdT expression was restricted to motor axons at some NMJs (Fig. 3*A*), the reporter fluorescence was present also in S100B^+^ Schwann cells at others and along the preterminal axons that innervate them (Fig. 3*B*). Schwann cells are derived from neural crest cells, which emerge from the dorsal most aspect of the neural tube and migrate to the periphery during development (Woodhoo and Sommer, 2008; Jessen and Mirsky, 2019). A majority of these help achieve saltatory conduction along peripheral axons by generating myelin sheaths. A subtype of Schwann cells found at NMJs, called terminal Schwann cells, do not form myelin but have been shown to help remodel neuromuscular synaptic morphology during development and after injury (Griffin and Thompson, 2008; YI Lee et al., 2016, 2017). We quantified the percentage of NMJs that exhibited tdTomato expression in terminal Schwann cells, and found that muscles of the hindlimbs (e.g., extensor digitorum longus and soleus) contained greater proportions of labeled Schwann cells compared with muscles of the upper body (e.g., sternomastoid and triangularis sterni; Fig. 3*C*). This observation mirrors and is consistent with the greater abundance of tdT^+^ glial cells in the caudal spinal cord (Fig. 1), suggesting that some caudalizing factor is potentially upstream of Hb9 activity early in neural tube development.

### Hb9 is expressed in neural progenitors in the embryonic mouse spinal cord

**Figure 4.**
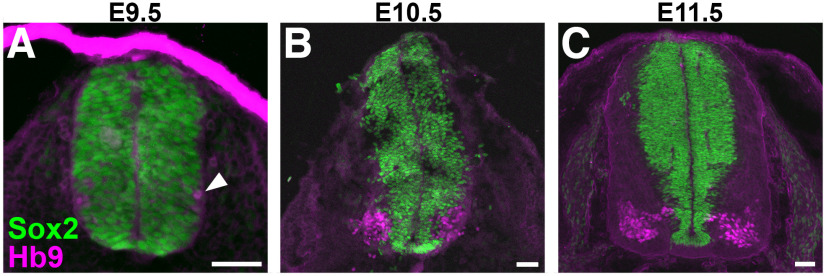
Hb9 expression in embryonic spinal cords. Transverse sections of lumbar spinal cord from (***A***) E9.5, (***B***) E10.5, and (***C***) E11.5 wild-type mouse embryos, with neural progenitor marker Sox2 labeled in green and Hb9 labeled in magenta. Arrowhead indicates emergence of Hb9^+^ cells at E9.5. Scale bars = 50 μm.

**Figure 5.**
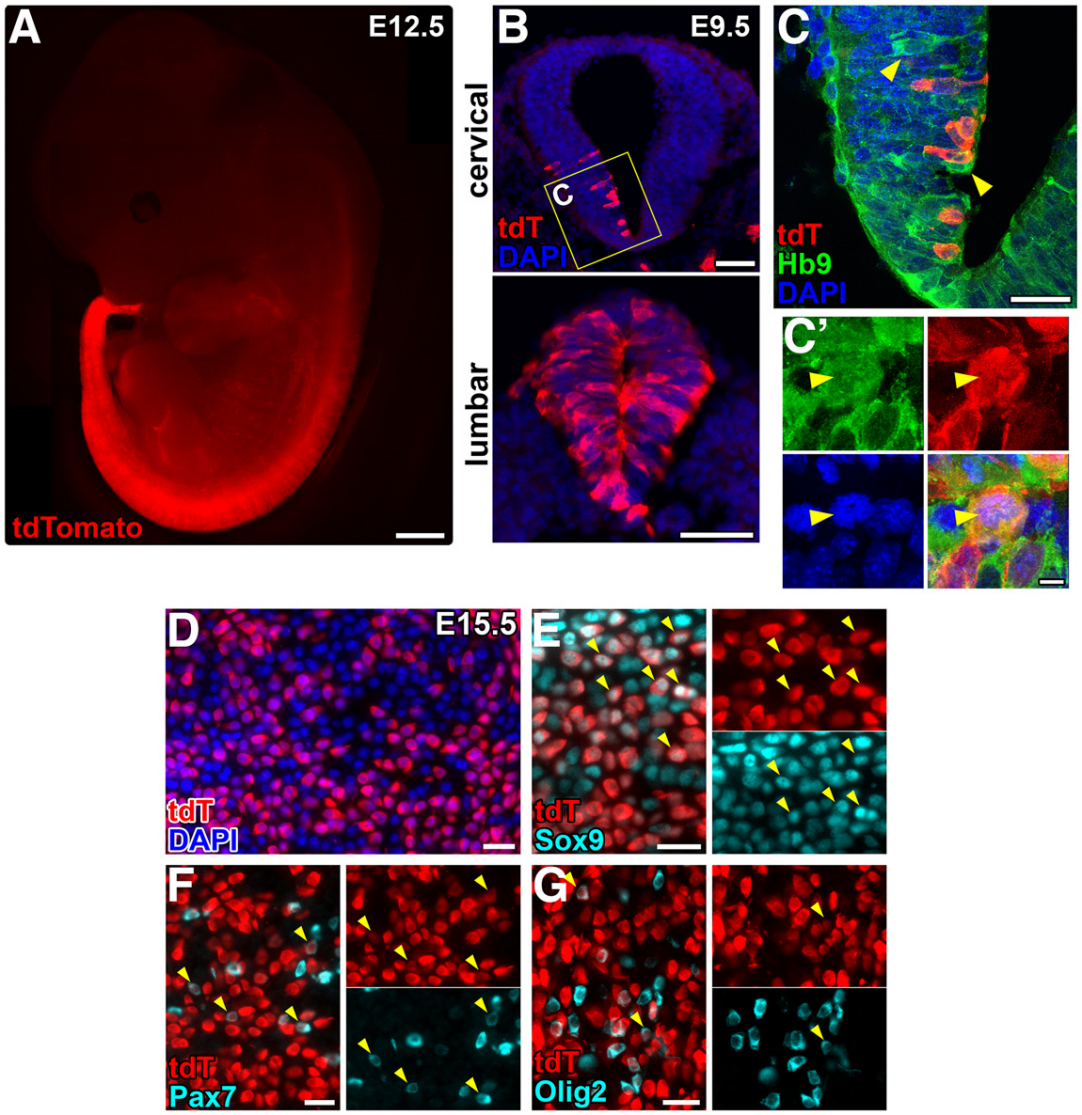
tdTomato is expressed in glial cells of the embryonic Hb9^cre^;Ai14 mouse in an increasing rostrocaudal gradient. ***A***, Fluorescent image of a whole E12.5 Hb9^cre^;Ai14 embryo. ***B***, Transverse sections of cervical and lumbar spinal cord from an E9.5 Hb9^cre^;Ai14 embryo. ***C***, ***C’***, Confocal images showing Hb9 immunoreactivity in the nuclei of some cells in the E9.5 lumbar spinal cord. ***C’***, Nuclear Hb9 expression in a few tdT^+^ cells (arrowheads). ***D–G***, Horizontal sections of lumbar spinal cord from E15.5 Hb9^cre^;Ai14 embryo. Arrowheads indicate tdT^+^ cells that express (***E***) Sox9, (***F***) Pax7, and (***G***) Olig2. Scale bars = 1 mm (***A***), 100 μm (***B***), 25 μm (***C*–*G***), and 5 μm (***C’***).

The observation that Hb9-lineage cells include glial cells as well as neurons in the adult mouse spinal cord suggests that Hb9 might be expressed in a subset of neural progenitor cells in the developing spinal cord. We assessed native patterns of Hb9 expression in the embryonic cervical spinal cord and found that Hb9 immunoreactivity is first detectable around E9.5, is expressed in greater numbers of cells by E10.5–E11.5, and does not overlap with the neural progenitor marker, Sox2 (Fig. 4). These findings are consistent with previous reports of Hb9 protein expression in the embryonic spinal cord as early as E9.5 in mice (Arber et al., 1999; Thaler et al., 1999). We examined tdT expression in embryonic Hb9^cre^;Ai14 mice and detected an increasing rostrocaudal gradient of tdT^+^ cells in the spinal cord (Fig. 5*A*), similar to the adult (Fig. 1*B*). This gradient was apparent as early as E9.5, with very few tdT^+^ cells present at rostral spinal cord levels compared with caudal levels (Fig. 5*B*). To determine whether tdTomato expression in the early Hb9^cre^;Ai14 spinal cord was potentially because of off-target Hb9 expression, we therefore assessed Hb9 immunoreactivity at E9.5. Hb9 was detected in the cytoplasm, but not nuclei, of tdTomato-negative cells in the E9.5 spinal cord (Fig. 5*C*), suggesting that Hb9 is expressed, but not transcriptionally active in these cells (Leotta et al., 2014). While most tdTomato-expressing cells in the spinal cord did not have detectable Hb9 immunoreactivity at this stage, we detected a few tdT^+^ cells (<1%) that had nuclear Hb9 immunoreactivity in the lumbar spinal cord (Fig. 5*C****’***). Together, these findings suggest that Hb9 activity is present in progenitor cells of the neural tube at stages earlier than E9.5, before MNs are born (Arber et al., 1999).

**Figure 6.**
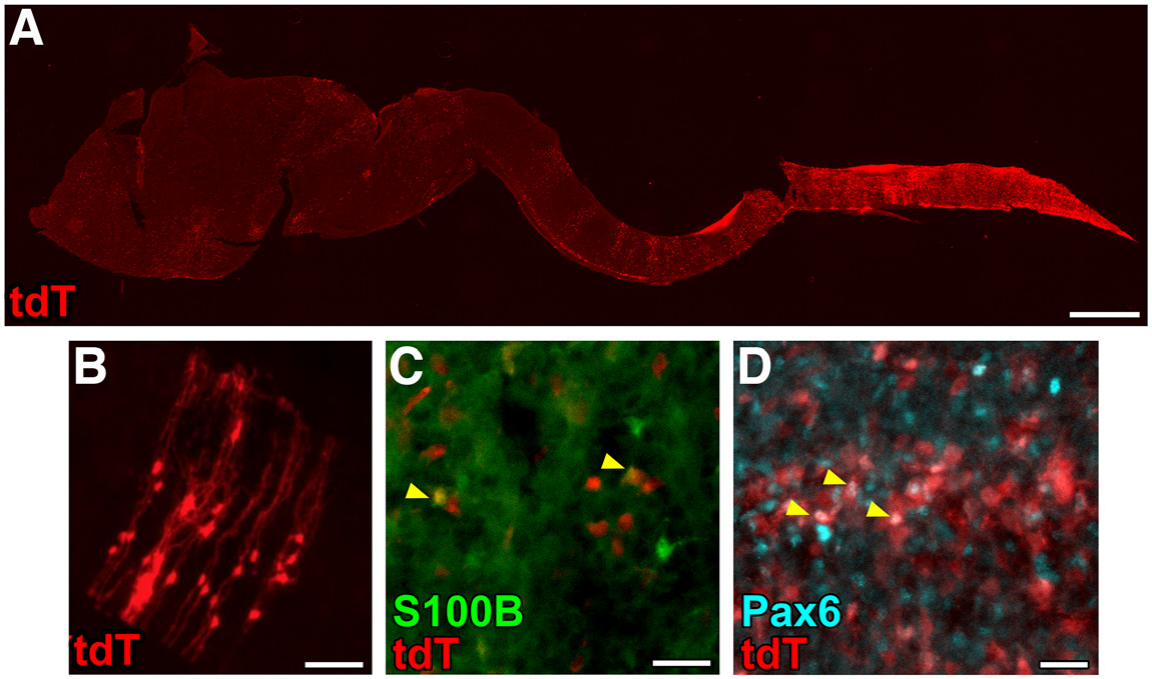
tdTomato is expressed in neural and glial progenitors in neonatal Hb9^cre^;Ai14 mice. ***A***, Sagittal section through the nervous system of a P0 Hb9^cre^;Ai14 mouse. ***B***, tdTomato^+^ cells with radial glia-like morphology in the spinal cord of a P0 Hb9^cre^;Ai14 mouse. ***C***, ***D***, tdTomato is expressed in a subset of (***C***) S100B^+^ cells and (***D***) Pax6^+^ cells in the neonatal spinal cord, indicated with arrowheads. Scale bars = 1 mm (***A***) and 50 μm (***B–D***).

We next sought to better characterize the populations of Hb9-lineage cells present in the embryonic lumbar spinal cord. At E15.5, neurogenesis in the spinal cord is largely complete and gliogenesis is underway (Lu et al., 2015; Lai et al., 2016). We detected tdTomato expression in large numbers of cells expressing Sox9, indicating glial (astrocyte and oligodendrocyte) identity (Stolt et al., 2003; Fig. 5*D*,*E*). In addition, we also identified tdTomato expression in a subset of cells expressing Pax7, a definitive marker of dorsal spinal cord NPCs including those that give rise to neural crest (Mansouri and Gruss, 1998; Murdoch et al., 2012; Roellig et al., 2017; Fig. 5*F*). We only identified a very small proportion of tdT^+^ cells that also expressed Olig2, a marker of pMN progenitors that give rise to MNs and oligodendrocytes (Novitch et al., 2001; SK Lee et al., 2005; Fig. 5*G*). Hence, in the Hb9^cre^;Ai14 mouse, Hb9-lineage cells include dorsal spinal cord cells and glial cells, consistent with our observations in the adult mouse (Fig. 1). This is further confirmed by immunostaining performed in the neonate (Fig. 6).

### Caudalizing morphogens promote gliogenesis and glial tdTomato expression in cultured spinal cord neural progenitor cells

**Figure 7.**
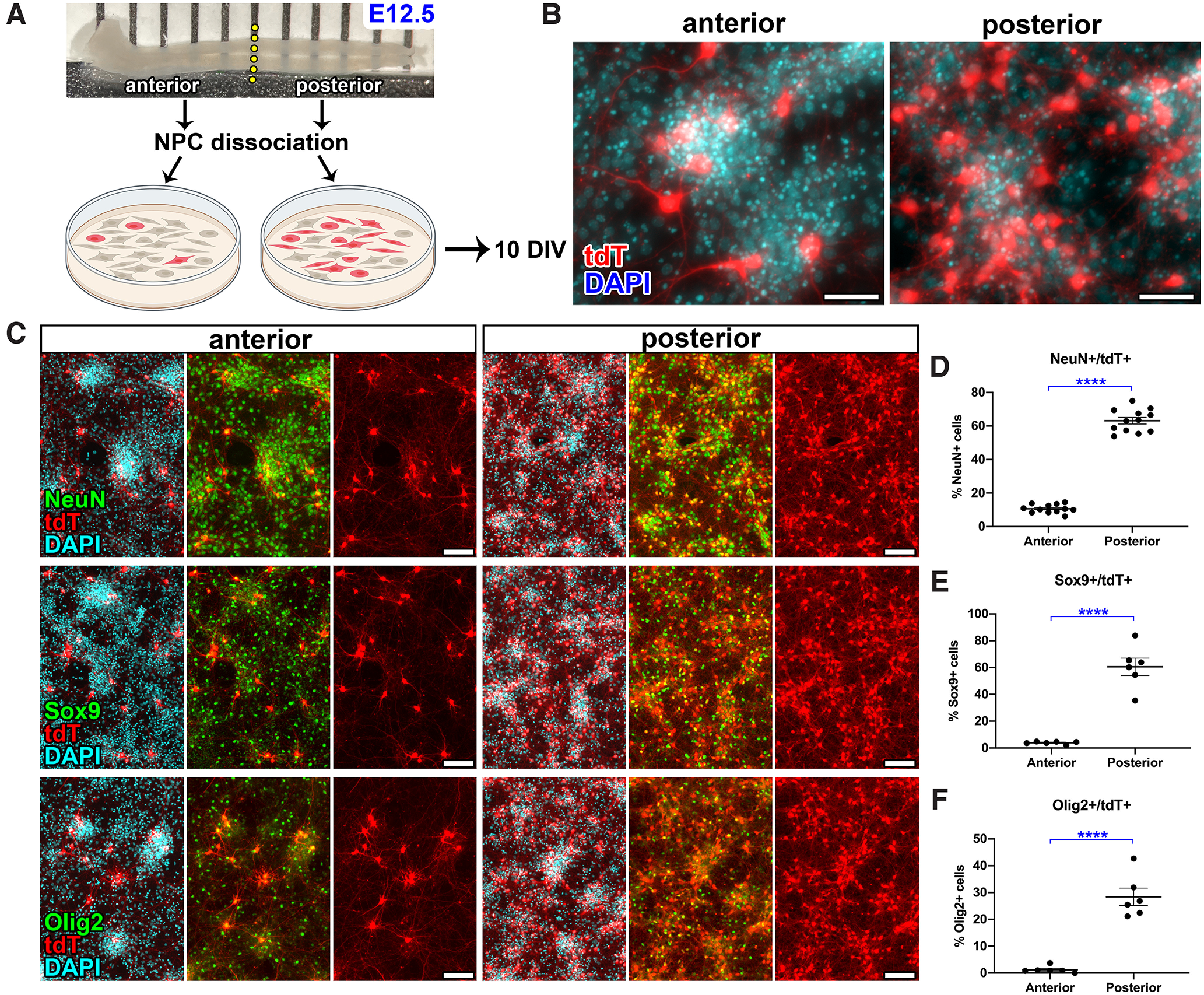
Cultured neural progenitor cells from Hb9^cre^;Ai14 embryonic spinal cord give rise to tdTomato^+^ neurons and glial cells. ***A***, Cartoon shows experimental design for data in panels. ***B–D***, Spinal cords from E12.5 Hb9^cre^;Ai14 embryos were separated into anterior and posterior halves. Tissue was dissociated, and neural progenitor cells (NPCs) obtained from either anterior or posterior spinal cord were cultured for 10 d *in vitro*. ***B***, Representative images of tdTomato expression in anterior or posterior NPC cultures after 10 d. ***C***, Expression of neuronal marker NeuN (top row), astroglial marker Sox9 (middle row), and oligodendroglial/motor neuron marker Olig2 (bottom row) in NPC cultures. ***D–F***, Quantification of the percent of (***D***) total NeuN^+^, (***E***) Sox9^+^, or (***F***) Olig2^+^ cells that express tdTomato. All data are mean ± SEM *N* = 6 per group. ****p < 0.0001. Scale bars = 50 μm (***B***) and 100 μm (***C***).

In normal development, morphogen gradients in the environment including Wnts, FGFs, and Gdf11 activate differential gene expression in spinal cord NPCs along the rostrocaudal axis (Liu et al., 2001; Nordström et al., 2002; Chanut, 2006; Liu, 2006). In order to identify putative factors that could modulate Hb9 expression in the spinal cord, we first established an *in vitro* assay conducive to drug treatment. The anterior or posterior halves of E12.5 Hb9^cre^;Ai14 spinal cord were isolated, dissociated, then cultured for 10 d (Fig. 7*A*). As expected, cultures of NPCs derived from the anterior spinal cord contained relatively few tdTomato^+^ cells compared with posterior NPCs (anterior: 3.00 ± 0.137%, posterior: 19.0 ± 1.13%, *p* < 0.0001; Fig. 7*B*). We analyzed the numbers of NeuN^+^ (neuron), Sox9^+^ (astrocyte/oligodendrocyte), and Olig2^+^ (MN/oligodendrocyte) cells expressing tdTomato and found that for each cell type, there were greater numbers of cells expressing tdTomato in posterior cultures versus anterior cultures (Fig. 7*C–F*). Thus, the rostrocaudal gradient of tdT^+^ cells is maintained even after removing neural progenitors from the environment of the developing spinal cord and culturing *in vitro*.

**Figure 8.**
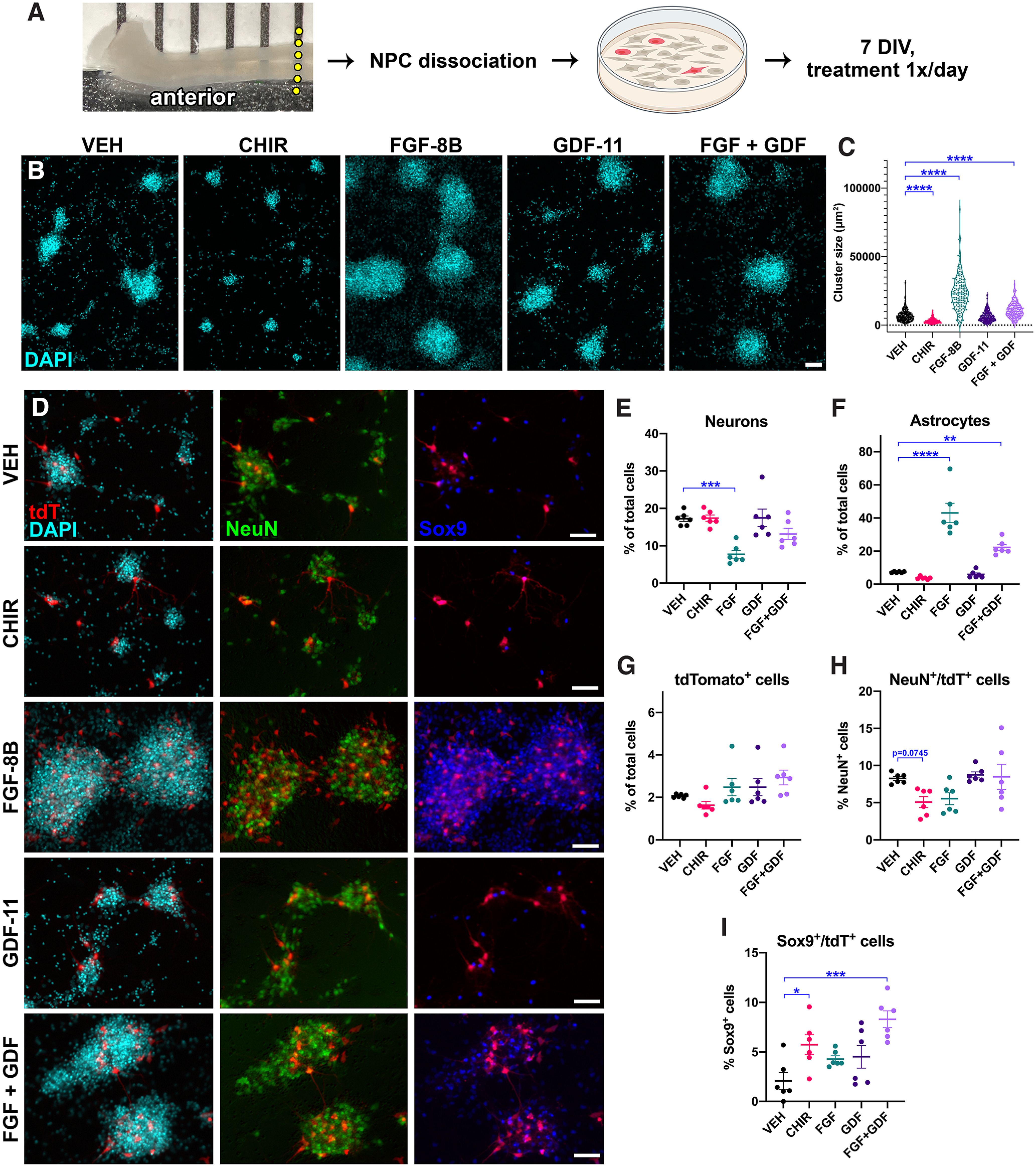
Treatment with caudalizing factors promotes gliogenesis and glial tdTomato expression in cultured NPCs. ***A***, Cartoon shows experimental design. Anterior portions of E12.5 Hb9^cre^;Ai14 spinal cords were dissociated, plated, and treated in culture for 7 d with either vehicle, CHIR 99 021, FGF-8B, GDF-11, or both FGF-8B + GDF-11. ***B***, Images of DAPI staining in cultured cells at 7 DIV. ***C***, Quantification of DAPI^+^ cell cluster size (μm^2^). ***D***, Representative images of cells in each treatment group immunolabeled for tdTomato, NeuN, and Sox9. ***E–I***, Quantification of (***E***) the percentage of all cells that are neurons, (***F***) the percentage of all cells that are astrocytes, (***G***) the percentage of all cells that are tdTomato^+^, (***H***) the percentage of neurons that express tdTomato, and (***I***) the percentage of astrocytes that express tdTomato. All data are mean ± SEM *N* = 6 per group. *p < 0.05, **p < 0.01, ***p < 0.001, ***p < 0.0001. Scale bars = 100 μm.

Using this *in vitro* assay, we next tested whether tdT expression in NPCs derived from the anterior E12.5 spinal cord could be induced by treatment with caudalizing factors (Fig. 8*A*). Beginning immediately after plating, and continuing once daily for 7 d, cells were treated either with the Wnt activator CHIR 99 021 (CHIR), FGF-8B, GDF-11, or both FGF-8B + GDF-11. Interestingly, we found that treatment with FGF alone or FGF+GDF significantly increased the size of cell clusters after 7 d *in vitro* (Fig. 8*B*,*C*). Cell density more than doubled in FGF-treated conditions (vehicle: 12,400 ± 763 cells/mm^2^, FGF-8B: 27,900 ± 2150 cells/mm^2^, *p* < 0.0001 by Student’s *t* test), suggesting that addition of FGF-8B promoted cell survival and/or increased proliferation. GDF-11 treatment had no effect on cell density or cluster size, whereas CHIR treatment significantly reduced cluster size (Fig. 8*B*,*C*) and cell density (CHIR: 9140 ± 1030 cells/mm^2^, *p* = 0.031 vs vehicle). We next evaluated the effects of treatment on neurogenesis and gliogenesis of cultured NPCs. Notably, we found that FGF treatment significantly reduced neuronal differentiation (Fig. 8*E*), and markedly increased glial differentiation (Fig. 8*F*). This suggests a progliogenic effect of FGF on spinal cord NPCs, similar to previous work showing that activation of FGF signaling induced astrocyte cell fates in the cerebral cortex (Dinh Duong et al., 2019). Despite the increased cell survival and/or proliferation on FGF treatment, we did not observe any differences in the percentage of total cells expressing tdTomato in any treatment group (Fig. 8*G*), nor did we observe any differences in the percentage of neurons that express tdT (Fig. 8*H*). However, we found that both CHIR and FGF+GDF treatment significantly increased the percentage of total astrocytes that expressed tdT (Fig. 8*I*). This observation that caudalizing factors increased reporter expression in glial cells suggests that combined FGF and GDF signaling may be implicated in the increased abundance of Hb9-lineage glial cells in the caudal spinal cord.

## Discussion

The Hb9^cre^ mouse has been used in hallmark studies to characterize the role of this transcription factor in differentiation of spinal MNs and consolidation of their identity (Arber et al., 1999). Recently, one study reported that YFP expression in Hb9^cre^;Rosa26-YFP mice was not only restricted to MNs, but also dorsal horn populations (Caldeira et al., 2017). However, the authors did not describe expression of reporter protein in glial cells of the spinal cord. Here, we present the surprising finding that Hb9-lineage cells include astrocytes and oligodendrocytes of the adult spinal cord, and Schwann cells in the peripheral nervous system. Our data suggest that Hb9 is expressed transiently in a population of cells, possibly neural progenitors, which give rise to neural crest and glial cells. These unexpected findings have implications for the design of experiments using the Hb9^cre^ mouse line. Indeed, genetic crosses made between the Hb9^cre^ mouse and mice with Cre-dependent alleles should be carefully monitored so that off-target effects are avoided. The use of an inducible Hb9^cre^ line (Koronfel et al., 2021), or viral vector-mediated gene expression instead of genetic crosses, might be better ways to achieve MN-specific manipulations in the mouse nervous system.

One important consideration of our study is whether the observed reporter expression patterns might be attributed to mosaicism. Mosaic patterns of recombination of Cre-dependent alleles can be a significant confounding factor in studies that use Cre driver mice for fate mapping. Indeed, the majority of Cre mouse lines have been shown to exhibit some degree of unreported recombinase activity, including mosaicism (Heffner et al., 2012). There are several lines of evidence suggesting that tdTomato expression in glial cells of Hb9^cre^;Ai14 mice is not because of mosaicism. First, we observed that this pattern of reporter expression is reproducible across multiple animals from different litters and consistently observed in all Hb9^cre^;Ai14 mice we analyzed. In addition, we identified identical patterns of gene expression in Hb9^cre^;Gq-DREADD mice (data not shown), suggesting that this is not an artifact related to the Ai14 mouse. In contrast, patterns of recombination because of mosaicism are unpredictable and inconsistent between littermates (Heffner et al., 2012). For this study, we only used male Hb9^cre^ sires, not female Hb9^cre^ dams, because maternal Cre expression has been shown to affect Cre excision patterns (Eckardt et al., 2004). Furthermore, *hb9:GFP* zebrafish embryos have been shown to exhibit a similar pattern of graded GFP expression that increases rostrocaudally in the developing spinal cord (Arkhipova et al., 2012).

Our descriptive study has several limitations. The most major limitation is that we were unable to detect Hb9 immunoreactivity in tdTomato^+^ cells, except for in a few cells in E9.5 embryos. We also failed to detect Hb9 transcript levels in these embryos (data not shown). Our results indicate that Hb9 expression occurs earlier than E9.5; however, because of technical limitations we did not assess earlier stages of development. Further work is needed to determine exactly when Hb9 is expressed in spinal cord progenitors, and these progenitors’ identities. Additionally, the exact mechanism by which Hb9-lineage cells become distributed in a gradually increasing rostrocaudal gradient is not clear. It is apparent that this gradient exists from as early as E9.5 in the spinal cord, which coincides with the very beginning of neurogenesis. This indicates that the graded distribution of Hb9-lineage cells is probably not because of migration, but potentially to gradients in environmental factors such as morphogens. Our *in vitro* results showed that addition of the caudalizing morphogen FGF-8B to cultured spinal cord neural progenitors was sufficient to promote massive gliogenesis at the expense of neurogenesis; moreover, addition of FGF-8B + GDF-11 increased the numbers of tdTomato^+^ glial cells in these cultures. It is interesting to note that the effects of FGF-8B on cell cluster size were partially mitigated by addition of GDF-11 to cell culture (Fig. 8*B*,*C*). GDF-11 is a member of the TGFβ superfamily, which includes BMPs and GDFs as well as other family members such as Nodal (Simoni-Nieves et al., 2019). Because BMPs can sometimes exert antagonistic effects on FGF signaling during normal development (Schliermann and Nickel, 2018), we speculate that GDF-11 may likewise antagonize proliferative effects of FGF in developing neural progenitor cells. Although we did not mechanistically demonstrate a link between FGF signaling and Hb9 transcriptional activity, our results collectively suggest that caudalizing morphogens may be upstream of glial Hb9 expression. Indeed, in studies describing the directed differentiation of embryonic stem cells *in vitro*, it has long been appreciated that caudalizing morphogens such as Sonic hedgehog are required for generation of motor neurons (Wichterle et al., 2002). Future work is needed to determine whether morphogen gradients in early development may induce rostrocaudal Hb9 expression in a concentration-dependent manner.

Our results also raise new, unanswered questions. First, it is unclear whether there are transcriptional and/or functional differences between Hb9-lineage glial cells and non-Hb9-lineage glial cells. Glial heterogeneity is an understudied but important topic in neurobiology, and transcriptionally distinct astrocyte populations have been shown to exist in different regions of the CNS (Tsai et al., 2012; Batiuk et al., 2020; Huang et al., 2020; Clarke et al., 2021). Future work could perform fluorescent-activated cell sorting on spinal cord tissue of Hb9^cre^;Ai14 mice and compare transcriptional profiles of Hb9-lineage versus non-Hb9-lineage astrocytes to determine these populations are transcriptionally diverse. Characterization of the differences between these cell types may reveal new functional roles for molecularly-distinct glial subtypes throughout the spinal cord. In addition, it would be interesting to test whether loss of Hb9 expression affected development of spinal cord glial cells. Finally, the distribution of molecularly-defined cell types in a gradually increasing rostrocaudal gradient is an intriguing and uncommon observation, and it is unclear exactly how this occurs. More work is needed to understand the mechanisms by which caudalizing morphogens induce transient Hb9 expression in the developing spinal cord.

## Synthesis

Reviewing Editor: Deanna Smith, University of South Carolina Decisions are customarily a result of the Reviewing Editor and the peer reviewers coming together and discussing their recommendations until a consensus is reached. When revisions are invited, a fact-based synthesis statement explaining their decision and outlining what is needed to prepare a revision will be listed below. The following reviewer(s) agreed to reveal their identity: NONE.

Both reviewers and I agree this is an interesting manuscript presents some intriguing novel findings based on an interesting approach to lineage tracing. However, the reviewers raised several concerns. One concern is related to problems with the Hb9 antibody. Also, some key controls should be added to further support the interpretations, and additional information on how some of the analyses were performed is needed. Finally, some additional analyses from tissues, rather than cells, would be informative and increase the potential impact of this work.

Specific concerns are outlined below.

Reviewer 1:

As the authors point out, a limitation of the manuscript is the inability to obtain co-staining of HB9 with the reporter, possibly due to the need to use very early embryos. There may not be HB9 expression in subsequent progeny of HB9 expressing cells, buth the authors could potentially be able to detect HB9 mRNA in early embryos and later progeny using in situ hybridization with a sensitive method such as RNAscope.

Since there seems to be an increase in the number of reporter-positive glia that are obtained during the culture period, I wonder whether the investigators might actually observe HB9 protein expression in glial cells or progenitors during the culturing period. This seems reasonable to try if they have not already.

Reviewer 2

Major Comments

Analysis of Cre(-) mice should be performed, to rule out leaky reporter expression or possible germline recombination

Additional information is needed regarding quantification of cells. How were ROIs identified to quantify cell cluster size to reduce or eliminate bias in the selection of cell clusters that were measured? Automated cell counting methods were validated by manual counts, but this is not described. How were manual counts performed for cell clusters and in tissues? How were regions selected for analysis?

It would be interesting to know what fraction of glial cells belong to the Hb9 lineage in adult spinal cord in vivo, and their distribution in the spinal cord. Are they localized predominantly to specific dorsal/ventral regions? The images in Figure 1 do not allow the reader to get a sense of their distribution.

Quantitative analysis of colocalization was performed with epifluorescent images instead of confocal images, creating uncertainty about true colocalization of signal vs. signal derived from different focal planes appearing to be colocalized, particularly given these sections were somewhat thick at 30μm and imaging was performed at using low power objectives. Confocal analysis would be more rigorous.

Figure 1 - the tdT labeled cells in E-F are identified as cells with astrocyte or oligo morphology, however this is difficult to discern from the image. As depicted, the tdT signal in E and F do not show typical astrocytic or oligo morphology and could reflect tdT signal from neuronal fibers. The double labeling with GFAP is not convincing as the GFAP signal does not appear to be in the same plane as the tdT signal and could instead reflect GFAP fibers in the vicinity of tdT labeled fibers from neurons or oligos. If these are confocal images, a single plane from a z-stack taken at higher magnification would more convincingly demonstrate colocalization of GFAP and tdT signal. The text describes quantification of astrocytes using Sox9, which is a more reliable marker for quantifying astrocytes than GFAP. It would be useful to demonstrate an example of the colocalization labeling between tdT and Sox9 used for analysis.

Figure 1 - The authors claim that tdT signal is restricted to ChAT neurons in cervical levels, however, there appears to be considerably more tdT signal panel 1C’ than ChAT-labeled cells. It would be useful to include a panel showing the overlay of the tdT and ChAT signal at all levels to enable readers to evaluate the labeling.

Colocalization between Hb9 and tdT in Figure 2C is difficult to discern. Single channel images showing only the Hb9 expression would be useful and more convincing than the orthogonal confocal image shown in panel 2C’.

Cytoplasmic vs. nuclear localization suggests Hb9 is expressed but not activated (?) what does this mean? Hb9 is a transcription factor

At E9.5, <1% of tdT cells showed nuclear Hb9-IR - what spinal cord level was analyzed? Panel B shows considerable tdT labeling in dorsal lumbar spinal cord, is there Hb9 expression at this level? It would be useful to analyze Hb9 expression at multiple ages

A better understanding of native expression of Hb9 at different ages and at different levels is arguably a key piece of information needed to better assess the fidelity of the Hb9 Cre line and evaluate the author’s conclusions that Hb9 lineage cells encompass cell types beyond the conventional understanding of Hb9 labeling primarily motor and interneurons. However, analysis and characterization of native Hb9 expression was limited to a single age and information on the spinal cord level analyzed was not included.

A major observation of the study is the differential distribution/abundance of Hb9 lineage cells across the rostro-caudal axis, however several analyses failed to identify or distinguish the specific level analyzed (e.g. Fig. 2C and C’, 2D-G

GDF-11 treatment alone has no effect on cell cluster size or density, but together with FGF shows a more limited increase in cell cluster size than FGF treatment alone. Why??? It would be useful for the authors to comment on this in the Discussion.

Minor Comments

The approximate level at which the anterior/posterior halves of spinal cord were transected for in vitro studies should be clarified (i.e., is this thoracic or lumbar, do anterior or posterior halves include partial lumbar or thoracic levels, respectively?)

Image quality of images in Figure 3B is very poor, and there appears to be quite a lot of background, particularly for an image of cells in culture. Individual cells are difficult to discern and colocalization is difficult to evaluate. A higher magnification image of a single cell cluster would facilitate evaluation of colocalization.

## Author Response

We thank the reviewers for their time and effort in reviewing our manuscript. We appreciate the positive feedback about our study, and we agree with the reviewers that our findings are intriguing and novel. We have carefully considered all of the reviewers’ comments and integrated suggested changes into the revised version of the manuscript. Please see below for a point-by-point response to reviewers’ comments (our responses are in blue). Note that we have highlighted any new changes/additions in the revised manuscript.

Reviewer 1:

As the authors point out, a limitation of the manuscript is the inability to obtain co-staining of HB9 with the reporter, possibly due to the need to use very early embryos. There may not be HB9 expression in subsequent progeny of HB9 expressing cells, buth the authors could potentially be able to detect HB9 mRNA in early embryos and later progeny using in situ hybridization with a sensitive method such as RNAscope.

At the reviewer’s suggestion, we have tried RNAscope to detect Hb9 transcript in frozen embryonic spinal cord sections (E9.5, E10.5, and E11.5), but unfortunately we failed to detect expression. We have now mentioned this in the Discussion.

Since there seems to be an increase in the number of reporter-positive glia that are obtained during the culture period, I wonder whether the investigators might actually observe HB9 protein expression in glial cells or progenitors during the culturing period. This seems reasonable to try if they have not already.

We did perform immunolabeling against Hb9 in the cultured cells, and failed to detect any Hb9 expression. The below image was taken of cells that were cultured 3 days in vitro. This lack of Hb9 immunoreactivity may be because this is too late for progenitor Hb9 expression (see above comment). Please note that we have verified this Hb9 antibody to work in embryonic spinal cord sections from E9.5-11.5 (see Figure 4).

Reviewer 2

Major Comments

Analysis of Cre(-) mice should be performed, to rule out leaky reporter expression or possible germline recombination.

We have now performed immunostaining on spinal cords of adult Ai14 mice (in the absence of any Cre), and confirmed that there is no tdTomato expression. We have added this data to Figure 2. In the first paragraph of the Results section, we added the following sentence: “We analyzed reporter expression in Ai14 mice (without Cre), and found a complete absence of tdTomato expression (Fig. 2), indicating that reporter expression is Cre-dependent, as expected.”

Additional information is needed regarding quantification of cells. How were ROIs identified to quantify cell cluster size to reduce or eliminate bias in the selection of cell clusters that were measured? Automated cell counting methods were validated by manual counts, but this is not described. How were manual counts performed for cell clusters and in tissues? How were regions selected for analysis?

We have now revised the Methods section under the “Image analysis” heading to address these questions. Changes are highlighted in the manuscript text:

"Watershed was applied to binary images and the Analyze Particles function was used to count the total number of cells in the entire image (no ROIs were drawn). Automated cell counting methods were always validated by manual counts for two images within each batch; two independent experimenters hand-counted cells in a blinded fashion, then hand counts were compared to results generated with the automated cell counting macro. We found that inter-experimenter counts were 99% accurate and >95% consistent with automated counts.”

"Quantification of cell cluster size: Regions of interest were manually drawn around contiguous clusters of DAPI+ cells in which all cells were touching each other, and the total area of each cluster was measured in ImageJ. ROIs were drawn around every cluster in all images, to avoid quantification bias.”

It would be interesting to know what fraction of glial cells belong to the Hb9 lineage in adult spinal cord in vivo, and their distribution in the spinal cord. Are they localized predominantly to specific dorsal/ventral regions? The images in Figure 1 do not allow the reader to get a sense of their distribution.

To address the reviewer’s first question of what fraction of glial cells belong to the Hb9 lineage in the adult spinal cord, we performed a new analysis to calculate the percentage of Sox9+ glial cells that express tdTomato at each spinal level, the results of which are now shown in Figure 1M. As expected, the total fraction of glial cells gradually increases in the caudal direction until the sacral spinal cord which has over 80% tdt+ glia. To answer the reviewer’s second question (“Are they localized predominantly to specific dorsal/ventral regions?”), we have performed a new analysis, the results of which are now shown in Figure 1J-L. Interestingly, we have found that at all spinal levels except cervical, tdTomato+ neurons and gray matter astrocytes are significantly enriched in the dorsal gray matter, while tdTomato+ white matter astrocytes are significantly enriched in the ventral white matter at sacral levels.

Quantitative analysis of colocalization was performed with epifluorescent images instead of confocal images, creating uncertainty about true colocalization of signal vs. signal derived from different focal planes appearing to be colocalized, particularly given these sections were somewhat thick at 30μm and imaging was performed at using low power objectives. Confocal analysis would be more rigorous.

We respectfully submit that we have used high-quality epifluorescent images for analysis in this study, and we are confident in the rigor of our colocalization analysis. (As a note to the reviewers, the quality of figures in the manuscript sent out for review appears to be greatly downsampled from the original resolution.) We respectfully disagree that labor- and time-intensive analysis of confocal images is required to provide confidence in our data.

Figure 1 - the tdT labeled cells in E-F are identified as cells with astrocyte or oligo morphology, however this is difficult to discern from the image. As depicted, the tdT signal in E and F do not show typical astrocytic or oligo morphology and could reflect tdT signal from neuronal fibers. The double labeling with GFAP is not convincing as the GFAP signal does not appear to be in the same plane as the tdT signal and could instead reflect GFAP fibers in the vicinity of tdT labeled fibers from neurons or oligos. If these are confocal images, a single plane from a z-stack taken at higher magnification would more convincingly demonstrate colocalization of GFAP and tdT signal. The text describes quantification of astrocytes using Sox9, which is a more reliable marker for quantifying astrocytes than GFAP. It would be useful to demonstrate an example of the colocalization labeling between tdT and Sox9 used for analysis.

We agree with the reviewer, and we have now replaced panel 1E with images of tdT+/Sox9+ astrocytes.

Figure 1 - The authors claim that tdT signal is restricted to ChAT neurons in cervical levels, however, there appears to be considerably more tdT signal panel 1C’ than ChAT-labeled cells. It would be useful to include a panel showing the overlay of the tdT and ChAT signal at all levels to enable readers to evaluate the labeling.

We now include an overlay of tdT and ChAT in Figure 1C.

Colocalization between Hb9 and tdT in Figure 2C is difficult to discern. Single channel images showing only the Hb9 expression would be useful and more convincing than the orthogonal confocal image shown in panel 2C’.

We have now done this. Please note that Figure 2C has become Figure 5C.

Cytoplasmic vs. nuclear localization suggests Hb9 is expressed but not activated (?) what does this mean? Hb9 is a transcription factor

We have changed the wording from “activated” to “transcriptionally active” for clarity.

At E9.5, <1% of tdT cells showed nuclear Hb9-IR - what spinal cord level was analyzed? Panel B shows considerable tdT labeling in dorsal lumbar spinal cord, is there Hb9 expression at this level? It would be useful to analyze Hb9 expression at multiple ages.

We have revised this statement to note that the lumbar spinal cord was analyzed. We agree that it would be useful to analyze Hb9 expression at multiple ages, but we respectfully submit that this would add considerable time, effort, and cost to the study.

A better understanding of native expression of Hb9 at different ages and at different levels is arguably a key piece of information needed to better assess the fidelity of the Hb9 Cre line and evaluate the author’s conclusions that Hb9 lineage cells encompass cell types beyond the conventional understanding of Hb9 labeling primarily motor and interneurons. However, analysis and characterization of native Hb9 expression was limited to a single age and information on the spinal cord level analyzed was not included.

We have now added Figure 4 to show native Hb9 expression in the lumbar spinal cord at E9.5-11.5.

A major observation of the study is the differential distribution/abundance of Hb9 lineage cells across the rostro-caudal axis, however several analyses failed to identify or distinguish the specific level analyzed (e.g. Fig. 2C and C’, 2D-G

We have now specified in the figure legends and results section the spinal levels that were analyzed.

GDF-11 treatment alone has no effect on cell cluster size or density, but together with FGF shows a more limited increase in cell cluster size than FGF treatment alone. Why??? It would be useful for the authors to comment on this in the Discussion.

The most straightforward explanation for this would be that the activity of GDF-11 is antagonistic to the activity of FGF-8B on cultured NPCs in vitro. We have added this to the Discussion: “It is interesting to note that the effects of FGF-8B on cell cluster size were partially mitigated by addition of GDF-11 to cell culture (Fig. 8B-C). GDF-11 is a member of the TGFβ superfamily, which includes BMPs and GDFs as well as other family members such as Nodal (Simoni-Nieves et al., 2019). Because BMPs can sometimes exert antagonistic effects on FGF signaling during normal development (Schliermann and Nickel, 2018), we speculate that GDF-11 may likewise antagonize proliferative effects of FGF in developing neural progenitor cells.”

Minor Comments

The approximate level at which the anterior/posterior halves of spinal cord were transected for in vitro studies should be clarified (i.e., is this thoracic or lumbar, do anterior or posterior halves include partial lumbar or thoracic levels, respectively?)

We have now clarified this in the Methods section under the heading “Embryo generation and neural progenitor cell isolation”:

"For in vitro studies, spinal cords were cut transversely at approximately 4.0-4.5 mm from the rostral end to separate the anterior and posterior segments. In this way, the “anterior” segment included the cervical and upper half of the thoracic cord, and the “posterior” segment included the lumbar and lower half of the thoracic cord.”

Image quality of images in Figure 3B is very poor, and there appears to be quite a lot of background, particularly for an image of cells in culture. Individual cells are difficult to discern and colocalization is difficult to evaluate. A higher magnification image of a single cell cluster would facilitate evaluation of colocalization.

(Please note that Figure 3B has now become Figure 7B.) We question if the reviewer’s perception of poor quality figures is due to the downsampling used in the manuscript review process. We have pasted the original figure below so the reviewer can appreciate the detail. Regardless, higher magnification images have now been used for Fig. 7B.
